# Simplified Fracture Mechanics Analysis at the Zinc–Adhesive Interface in Galvanized Steel–CFRP Single-Lap Joints

**DOI:** 10.3390/ma18215038

**Published:** 2025-11-05

**Authors:** Maciej Adam Dybizbański, Katarzyna Rzeszut

**Affiliations:** Faculty of Civil and Transport Engineering, Poznań University of Technology, 5 Marii Skłodowskiej-Curie Str., 60-965 Poznań, Poland; maciej.a.dybizbanski@gmail.com

**Keywords:** adhesive joints, galvanized steel, interface fracture mechanics (IFM), energy release rate, single-lap joint

## Abstract

Adhesively bonded joints between galvanized steel and carbon fiber-reinforced polymers (CFRPs) are critical in modern lightweight structures, but their performance is often limited by failure at the zinc–adhesive interface. This study presents a parametric analysis to investigate the influence of key geometric parameters on interfacial cracking in a single-lap joint (SLJ) configuration, employing a simplified analytical methodology based on Interface Fracture Mechanics (IFM). The model combines the Goland–Reissner approach for estimating crack-tip loads with highly simplified, constant shape functions to calculate the energy release rate ($G_{int}$) and phase angle (ψ). To provide a practical reference, experimental data from shear tests on S350 GD galvanized steel bonded to CFRP were used to estimate the range of interfacial fracture toughness for this material system. The parametric results demonstrate that, for a constant load, increasing the overlap length reduces the crack driving force ($G_{int}$), while increasing the adhesive thickness raises it. Crucially, the model indicates that a thicker adhesive layer shifts the fracture mode from shear- to opening-dominated, a trend consistent with the established mechanics of SLJs, where increased joint rotation amplifies peel stresses. The study concludes that while the use of constant shape functions limits the model’s quantitative accuracy, this simplified analytical framework effectively captures the qualitative influence of key geometric parameters on the joint’s fracture behavior. It serves as a valuable and resource-efficient tool for preliminary design explorations and for interpreting experimentally observed failure trends in galvanized steel–CFRP joints.

## 1. Introduction

Adhesive bonding is increasingly recognized as an effective method for joining diverse structural materials, including steel with carbon fiber-reinforced polymers (CFRPs) [1,2,3], especially in Rzeszut et al. [4]. The application of CFRP to reinforce or connect steel elements offers numerous advantages, such as mass reduction, increased load-bearing capacity, and improved fatigue and corrosion resistance [5,6]. However, designing durable and reliable joints of this type, particularly involving galvanized steel, presents several engineering challenges [7]. The zinc layer, applied to protect steel from corrosion [8,9], introduces an additional material interface (zinc–adhesive) that can become a site for damage initiation [10,11,12,13]. Understanding the failure mechanisms at this specific interface and the influence of various structural and material parameters on its behavior is crucial for ensuring the integrity of the entire joint [14,15].

Interface Fracture Mechanics (IFM) provides theoretical tools for analyzing cracks located at the boundary between two dissimilar materials [16,17,18]. This approach allows for the quantification of conditions for crack initiation and propagation through parameters such as stress intensity factors ($K_{1},K_{2}$), energy release rate ($G_{int}$), and phase angle (ψ), which describes the mixity of fracture modes [19,20]. Accurate IFM analyses are often complex and require advanced numerical methods, such as the finite element method employing special techniques for extracting fracture parameters [21]. However, there is a need to develop simplified analytical models that, despite certain approximations, would allow for effective parametric studies and preliminary assessment of joint behavior.

A critical challenge in the application of adhesively bonded steel–CFRP systems is the prevention of premature failure, with debonding at the material interface being recognized as a primary and critical failure mode [22]. The analysis of such failures is complex, as crack propagation along the interface typically occurs under mixed-mode loading conditions, involving a combination of normal (Mode I) and shear (Mode II) stresses [23]. Significant research has been dedicated to understanding this phenomenon. For instance, Zeng et al. (2018) [24] provided detailed investigations into the interfacial behavior and debonding mechanisms in full-scale CFRP-strengthened steel beams, highlighting the importance of fracture mechanics in predicting joint performance [24]. The field continues to evolve, with recent studies exploring the fracture characteristics of various bi-material combinations, including the Mode I tensile fracture behavior of metal–CFRP joints [25]. This body of work underscores the necessity of applying fracture mechanics principles to accurately characterize the interfacial behavior and ensure the long-term reliability of these hybrid structures.

Experimental studies on adhesively bonded galvanized steel–CFRP joints, such as those conducted by Dybizbański et al. (2024) [26], provide valuable data on the failure mechanisms and load-bearing capacities of these systems. In the cited work, various failure modes were observed, including failure at the steel–adhesive interface (interpreted here as zinc–adhesive), fiber rupture, and mixed-mode failures. It was also noted that an increase in adhesive thickness led to a decrease in joint strength, while an increase in overlap length generally improved the load-bearing capacity.

The motivation for the present work is the need for a systematic analysis of the influence of key geometric and material parameters on the conditions for crack initiation at the zinc–adhesive interface, using an analytical tool that is less resource-intensive than full numerical modelling. Such a parametric study, despite its inherent simplifications, can provide important insights into the sensitivity of the joint to design changes and aid in the interpretation of experimental results.

The main aim of this work is to conduct a parametric study of the influence of selected geometric parameters (overlap length L; adhesive layer thickness $t_{k}$; initial crack length a) on the energy release rate $G_{int}$ and phase angle ψ at the zinc–adhesive interface in steel–CFRP single-lap joints (SLJs), using a simplified analytical methodology based on IFM.

## 2. Theoretical Background

### 2.1. Interface Fracture Mechanics (IFM)–Key Parameters

Interface Fracture Mechanics (IFM) describes the behavior of cracks located at the boundary of two dissimilar materials. In the context of this work, material 1 is the zinc layer on the steel surface, and material 2 is the epoxy adhesive.

The stress field near an interfacial crack tip is characterized by a complex stress intensity factor $K=K_{1}+iK_{2}$ [16,27]. The components $K_{1}$ and $K_{2}$ correspond to the conventional Mode I (opening) and Mode II (in-plane shear).

The mismatch in elastic properties between material 1 (zinc) and material 2 (adhesive) is described by the Dundurs parameters $\alpha_{D}$ i $\beta_{D}$ [28,29] and the bimaterial constant ϵ (oscillation index) [16]:

(1) $$\alpha_{D}=\frac{{\bar{E}}_{1}-{\bar{E}}_{2}}{{\bar{E}}_{1}+{\bar{E}}_{2}}$$

(2) $$\beta_{D}=\frac{\mu_{1}\left(\kappa_{2}-1\right)-\mu_{2}\left(\kappa_{1}-1\right)}{\mu_{1}\left(\kappa_{2}+1\right)-\mu_{2}\left(\kappa_{1}+1\right)}$$

(3) $$\epsilon =\frac{1}{2\pi }\ln\left(\frac{\frac{\kappa_{1}}{\mu_{1}}+\frac{1}{\mu_{2}}}{\frac{\kappa_{2}}{\mu_{2}}+\frac{1}{\mu_{1}}}\right)$$

where ${\bar{E}}_{j}=E_{j}/(1-\nu_{j})$ for plane strain (PS), $\mu_{j}$ is the shear modulus, and $\kappa_{j}=3-4\nu_{j}$ for PS.

The energy release rate for an interfacial crack $G_{int}$ is related to $K_{1}$ i $K_{2}$ by $G_{int}=\left(K_{1}^{2}+K_{2}^{2}\right)/E_{eff}^{*}$ [16,20], where the effective Young’s modulus for the interface is calculated as follows [17]:

(4) $$E_{eff}^{*}={\left(\frac{\frac{1}{{\bar{E}}_{1}}+\frac{1}{{\bar{E}}_{2}}}{2}\right)}^{-1}$$

The relative proportion of fracture modes is characterized by the phase angle ψ, defined with respect to a characteristic length $L_{0}$ [16,17]:

(5) $$\psi =\mathrm{atan}2\left(Im\left(KL_{0}^{i\epsilon }\right),\;Re\left(KL_{0}^{i\epsilon }\right)\right)$$

In this work $L_{0}$ is taken as the adhesive layer thickness $t_{k}$.

**Figure 1:**
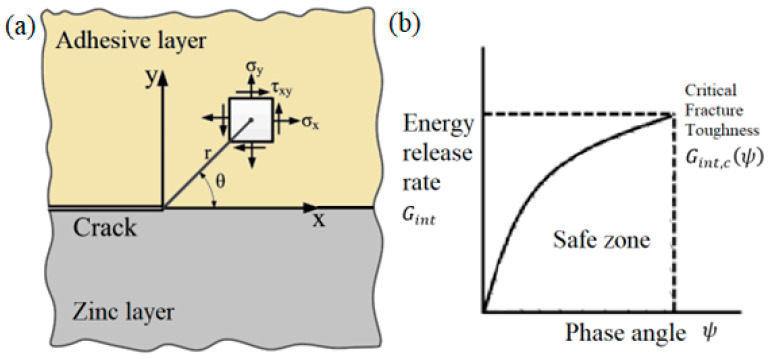
Key concepts of Interface Fracture Mechanics: (**a**) Stress field at a crack tip on the zinc–adhesive interface; (**b**) Energetic fracture criterion (fracture locus) showing the interfacial fracture toughness as a function of the phase angle [30].

Interfacial Fracture Toughness $G_{int,c}\left(\psi \right)$ is the critical value of $G_{int}$ required for crack propagation, which generally depends on the phase angle ψ [11,17]. Figure 1 presents those key concepts of IFM.

### 2.2. Goland–Reissner Model for Single-Lap Joints

The Goland–Reissner model [9,31] is a classical analytical model used to determine the stress distribution in the adhesive layer and the forces and moments in the adherends of lap joints, accounting for the eccentricity of the applied load. This leads to rotation of the overlap and induces bending moments in the adherends, as well as peel (normal) and shear stresses in the adhesive.

In this work, to estimate the bending moment $M_{edge}$ (per unit width) in the steel adherend at the edge of the overlap, the following simplified formula is adopted:

(6) $$M_{edge}=k^{\prime }\frac{Pt_{a}}{2}$$

where P is the force per unit width of the joint, and $t_{a}$ is the steel adherend thickness. The coefficient $k^{\prime }$ accounts for the adhesive flexibility and joint geometry; for the analysis, an approximate form is calculated as follows:

(7) $$k^{\prime }=\frac{1}{1+2\tanh\left(\beta_{shr}c\right)}$$

(8) $$\beta_{shr}=\sqrt{\frac{2G_{k}}{E_{a}t_{a}t_{k}}}$$

(9) $$c=\frac{L}{2}$$

where L is the overlap length, $E_{a}$ is Young’s modulus of steel, and $G_{k}$ is the shear modulus of the adhesive.

### 2.3. Simplified Shape Functions for Stress Intensity Factors

To calculate the stress intensity factors $K_{1}$ and $K_{2}$ for an edge crack at an interface, subjected to stresses $\sigma_{avg}$ (average axial stress in the adherend) and $\sigma_{bend}$ (bending stress in the adherend), the following general form is used [32,33]:

(10) $$K_{1}=\sigma_{\mathrm{avg}}\sqrt{\pi aF_{1N}}+\sigma_{bend}\sqrt{\pi aF_{1M}}$$

(11) $$K_{2}=\sigma_{avg}\sqrt{\pi aF_{2N}}+\sigma_{bend}\sqrt{\pi aF_{2M}}$$

where a is the crack length. In this work, to achieve maximum simplification of the analytical methodology for the parametric study, the following very general, constant values for the shape functions are adopted:

- $F_{1N}=1.12$: This value is a classical solution for the Mode I stress intensity factor for an edge crack in a semi-infinite homogeneous plate subjected to uniform tension, for $\frac{a}{t_{a}}\to 0$ [32,33].
- $F_{2N}=0$: This assumption arises from the fact that, in a homogeneous material, a symmetric tensile load on an edge crack does not induce Mode II.
- $F_{1M}=1.0$: For an edge crack in a homogeneous material subjected to bending, the Mode I shape function depends on $a/t_{a}$ [32]. The value of 1.0 is adopted as a further simplification for short cracks.
- $F_{2M}=0.3$: This value is taken as an estimate to account for a small potential Mode II component induced by the bending of an edge crack, particularly at an interface [17,34].

It must be strongly emphasized that these constant shape function values are a significant simplification and do not reflect the complex dependence on the relative crack length $\left(a/t_{a}\right)$ or the interface material mismatch parameters $\left(\alpha_{D},\beta_{D}\right)$. They represent a compromise between accuracy and the goal of creating a maximally simple analytical methodology suitable for a parametric study. Their impact on the results and limitations will be discussed later in the paper.

It should be emphasized that this work focuses on the energy release rate ($G_{int}$) and the phase angle (ψ), rather than on the individual stress intensity factors ($K_{1}$, $K_{2}$). For cracks at bimaterial interfaces, the near-tip stress fields exhibit an oscillatory character, which makes the separation of $K_{1}$ and $K_{2}$ non-unique and dependent on an arbitrarily chosen length scale. The analytical framework based on the ($G_{int}$, ψ) pair was developed to overcome this ambiguity, providing a more physically robust and unambiguous description of the crack driving force ($G_{int}$) and the mode mixity (ψ). It is therefore the preferred, modern approach for analyzing interfacial crack problems.

## 3. Methodology

### 3.1. Analyzed Joint Description and Model Idealizations

**Figure 2:**
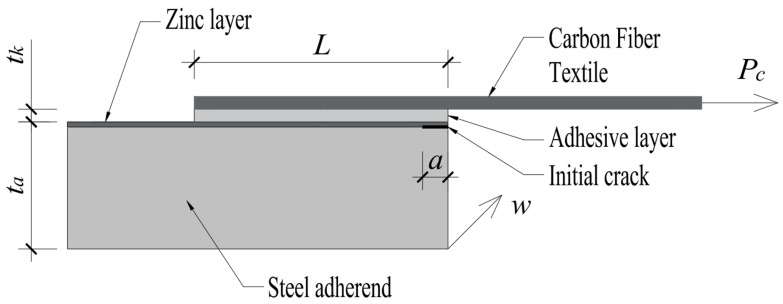
Schematic of the analyzed single-lap joint (SLJ), illustrating the key geometric parameters. The joint comprises a steel adherend (thickness $t_{a}$), an adhesive layer (thickness $t_{k}$), and a CFT adherend, with L denoting the overlap length, a the initial interfacial crack length and w joint width.

The analysis focuses on a single-lap joint (SLJ) with geometry and material characteristics corresponding to the specimens tested by Dybizbański et al. (2024) [26]. SLJ geometry is presented in Figure 2.

The joint consists of a steel adherend (S350 GD, thickness $t_{a}=2\;\mathrm{mm}$), the surface of which is coated with a zinc layer (thickness $t_{zinc}=0.009\;\mathrm{mm}$), an epoxy adhesive layer (SikaDur 330, thickness $t_{k}$), and a carbon fiber-reinforced polymer (CFRP SikaWrap 230 C) adherend. Both SikaWrap 230 C and SikaDur 330 were provided by Sika Poland sp. z o.o. (Warsaw, Poland).

An initial short crack of length a is assumed to be located at the interface between the zinc layer and the adhesive, at one end of the overlap. The crack length a is one of the parameters in the analysis. All materials (steel, zinc, adhesive) are treated as linear elastic and isotropic. The mechanical properties of zinc $\left(E_{zinc},\mu_{zinc,}\right)$ and the adhesive $\left(E_{k},\mu_{k}\right)$ are crucial for determining the interface parameters. The overlap length is denoted as L=2c, and the joint width as w.

Consistent with previous considerations, in the global SLJ analysis using the Goland–Reissner model, the influence of the zinc layer thickness on the global stiffness of the steel adherend is neglected, and $t_{a}=2\;\mathrm{mm}$ is used as the steel thickness. For the local IFM analysis at the zinc–adhesive interface, the mechanical properties of zinc are used to define the Dundurs parameters. In the arguments of the shape functions $F(a/t_{a},\alpha_{D},\beta_{D})$, $t_{a}=2\;\mathrm{mm}$ is used as the dimension characterizing the adherend whose stresses generate the loading at the crack tip located on the very thin zinc layer.

### 3.2. Calculation Steps of the Analytical IFM Model

The calculation procedure for IFM parameters for a given external force $P_{c}$ (over the entire joint width) is performed in four steps. Step 1 includes the calculation of elastic parameters for the zinc–adhesive interface. Based on the properties of zinc (material 1) and adhesive (material 2), for an assumed plane strain (PS) condition, the following are calculated: ${\bar{E}}_{zinc},{\bar{E}}_{adhesive},\mu_{zinc},\mu_{adhesive},\kappa_{zinc},\kappa_{adhesive},\alpha_{D},\beta_{D},\epsilon$ and $E_{eff}^{*}$, according to the formulas presented in point 2.1. In step 2 the estimation of loads $N_{a}(a),M_{a}(a)$ using the Goland–Reissner model are carried out for the force per unit width P=P_c/w, the bending moment $M_{edge}$ at the overlap edge in the steel adherend is calculated using the formula $M_{edge}=k^{\prime }Pt_{a}/2$ with the adopted form for k′. Then, applying simplifications, $N_{a}(a)\approx P$ and $M_{a}(a)\approx M_{edge}$ are assumed as the axial force and bending moment (per unit width) in the steel adherend cross-section at the crack tip. The calculation of $K_{1}$ and $K_{2}$, using simplified shape functions, are performed in step 3. Then, based on $N_{a}(a)$ and $M_{a}(a)$, the $\sigma_{avg}=N_{a}(a)/t_{a}$ and $\sigma_{bend}=6M_{a}(a)/t_{a}^{2}$ are calculated. Next, using the adopted constant shape function values ($F_{1N}=1.12$, $F_{2N}=0$, $F_{1M}=1.0$, $F_{2M}=0.3$), $K_{1}$ and $K_{2}$ are calculated according to the formulas in Section 2.3. Finally in step 4 the calculation of $G_{int}\;\mathrm{and}\;i\psi$ are performed. The energy release rate $G_{int}$ and the phase angle ψ (with $L_{0}=t_{k}$) are calculated based on $K_{1}$, $K_{2}$, $E_{eff}^{*}$ and ϵ according to the formulas in Section 2.1.

### 3.3. Parametric Study Plan

A parametric study was conducted to investigate the influence of key geometric parameters on the calculated $G_{int}$ and ψ values. The parameters as reference values for material and geometric properties were adopted based on Dybizbański et al. (2024) [26] and general material data. The joint width w was taken from the experimental specimens. The varied parameters that were taken into account include the following: overlap length L, adhesive layer thickness $t_{k}$, and initial crack length a. For each parametric analysis (varying one parameter while keeping others at baseline values), calculations of $G_{int}$ and ψ were performed for a constant, reference external force $P_{c}$. The choice of this force (e.g., corresponding to the average failure load from one of the experimental series [26] or a typical design load) allows for direct comparison of the parameter’s influence on the crack driving force. The primary output quantities analyzed were $G_{int}$ and ψ.

### 3.4. Processing of Experimental Data for $G_{int,c}\left(\psi \right)$ Estimation

Independently of the parametric study, experimental data concerning failure loads $P_{c,exp}$ from Dybizbański et al. (2024) [26] were processed using the described analytical methodology (Steps 1–4 from Section 3.2). For each specimen that underwent failure interpreted as adhesive at the zinc–adhesive interface, a pair ($G_{int},\psi$) was calculated. The resulting set of points was used to attempt an estimation of a segment of the interfacial fracture toughness curve $G_{int,c}\left(\psi \right)$ for the studied material system.

## 4. Results

### 4.1. Estimation of the Interfacial Fracture Toughness Curve Segment, $G_{int,c}\left(\psi \right)$, from Experimental Data

**Figure 3:**
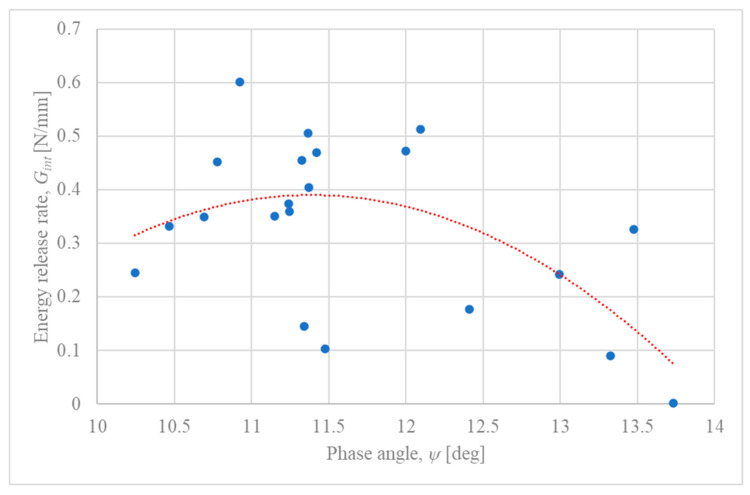
Estimation of the experimental interfacial fracture toughness locus for the S350 GD galvanized steel/SikaDur 330 adhesive system from experimental data [26]. Each point represents the calculated ($G_{int}$, ψ) pair at the measured failure load for specimens with interfacial failure. The dotted line represents a polynomial trendline, suggesting a non-linear dependence of fracture toughness on the phase angle.

Experimental failure load data ($P_{c,exp}$) for single-lap joints (SLJs) composed of S350 GD galvanized steel, SikaDur 330 adhesive, and SikaWrap 230 C carbon fiber textile, as reported by Dybizbański et al. (2024) [26], were processed using the analytical IFM model (Section 3.2). For each specimen that exhibited failure at the steel–adhesive interface (interpreted as the zinc–adhesive interface), a pair of ($G_{int},\psi$) values was calculated. The resulting data points are plotted in Figure 3.

As observed in Figure 3, the calculated phase angles (ψ) for the experimental failure points fall within a range of approximately 10° to 14°. While the individual data points show considerable scatter, varying from approximately 0.1 N/mm to over 0.5 N/mm, the addition of a polynomial trendline (red dotted line) reveals an underlying non-linear relationship. The trend suggests that the interfacial fracture toughness ($G_{int}$) is dependent on the phase angle, reaching a maximum value of approximately 0.4 N/mm at an optimal phase angle of around ψ≈11.5°. For phase angles deviating from this optimum, the toughness appears to decrease. This parabolic trend is consistent with the theoretical framework of Interface Fracture Mechanics, although it is partially obscured by the significant experimental variability, which can be attributed to factors discussed in Section 5.2.

### 4.2. Influence of Overlap Length (L) on IFM Parameters

**Figure 4:**
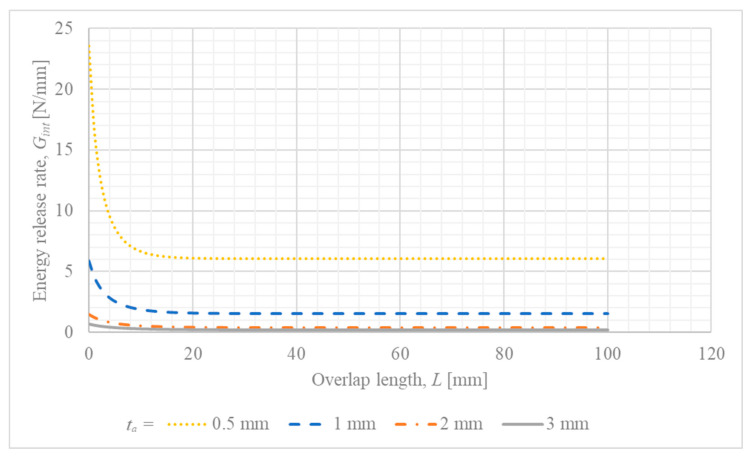
Influence of overlap length (L) on the energy release rate ($G_{int}$) for different steel adherend thicknesses ($t_{a}$).

To investigate the effect of overlap length (L) on the crack driving force, calculations of $G_{int}$ and ψ were performed for a continuous range of L values, from 0 mm to 100 mm. For this analysis, other parameters were kept constant at their baseline values, and a constant reference applied load $P_{c}$ was used to isolate the effect of the overlap length. The results for the energy release rate, $G_{int}$, for different thickness of the steel adherend $t_{a}$ are presented in Figure 4.

One can notice that for increased thickness of the steel adherend, the energy release rate is minimized for the whole considered range of overlap length. Another interesting observation is that the energy release rate becomes constant for overlap lengths greater than 20 mm.

### 4.3. Influence of Adhesive Layer Thickness ($t_{k}$) on IFM Parameters

The thickness of the adhesive layer is a critical parameter known to significantly influence the mechanical performance and failure behavior of adhesively bonded joints. To investigate this effect within the framework of the developed analytical model, a parametric study was conducted where the adhesive thickness ($t_{k}$) was varied from 0 to 1.0 mm. For this analysis, other parameters were held constant at their baseline values (L=15 mm, a=0.5 mm), and a constant reference applied load $P_{c}$ was used to isolate the influence of $t_{k}$ on the Interfacial Fracture Mechanics parameters.

**Figure 5:**
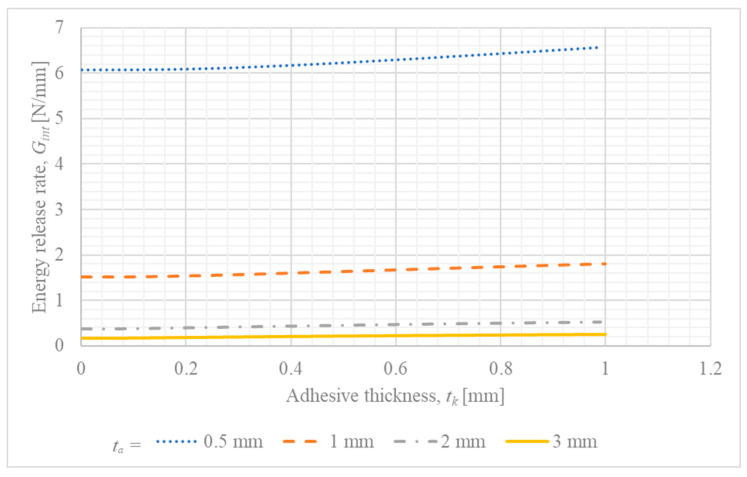
Influence of adhesive layer thickness ($t_{k}$) on the energy release rate ($G_{int}$) for different steel adherend thicknesses ($t_{a}$).

The results of this analysis are presented in Figure 5 ($G_{int}$ vs. $t_{k}$).

In the case of the investigation of the influence of adhesive thickness on energy release rate, the increased thickness of the steel adherend caused the reduction in energy release rate for the whole considered range of overlap length. Moreover, the energy release rate becomes almost constant for a large thickness of the steel adherend, and for the small, the result are of a nonliteral form.

**Figure 6:**
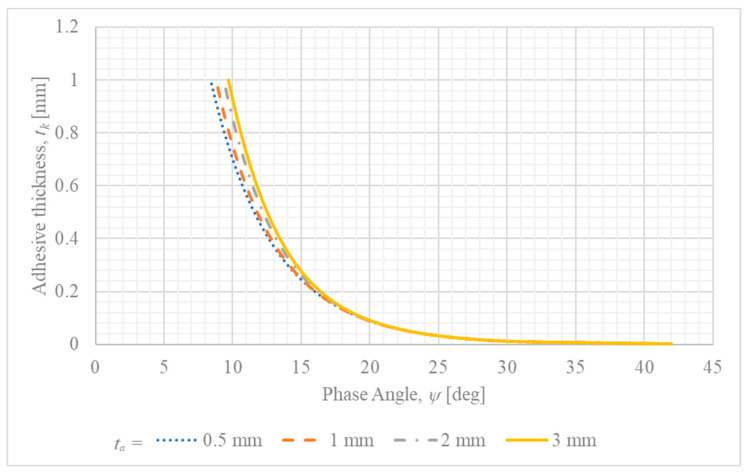
Dependence of the phase angle (ψ) on the adhesive layer thickness ($t_{k}$) for different steel adherend thicknesses ($t_{a}$).

Figure 6 presents phase angle–adhesive thickness relationships (ψ vs. $t_{k}$) for different adherend thicknesses.

It suggests that the increase in the adhesive layer thickness changes the fracture mode from shear-dominated to opening-dominated. Moreover, an increase in adherend thickness escalates this effect.

### 4.4. Influence of Assumed Initial Crack Length (a) on IFM Parameters

In any analysis based on Linear Elastic Fracture Mechanics (LEFM), it is necessary to assume the presence of an initial crack or flaw. The size of this assumed crack, a, can significantly influence the calculated fracture mechanics parameters. To assess the sensitivity of the developed analytical model to this parameter, a study was conducted where a was varied from a very small value (0.01 mm) up to 1.0 mm, while other parameters were held constant (L=15 mm, $t_{k}=0.5\;\mathrm{mm}$, and a reference applied load $P_{c}$).

**Figure 7:**
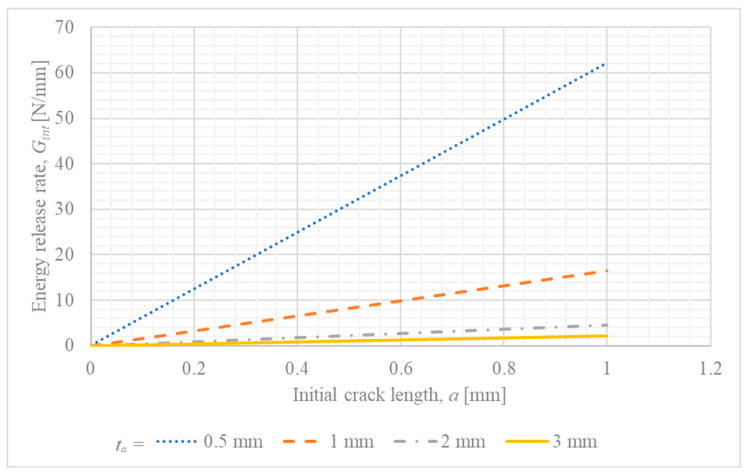
Sensitivity of the calculated energy release rate ($G_{int}$) to the assumed initial crack length (a) for different steel adherend thicknesses ($t_{a}$).

The results of this analysis are presented in Figure 7 ($G_{int}$ vs. a).

Based on the conducted analysis, it can be noticed that for all thicknesses of the steel adherend, the energy release rate remains proportional to the crack size. Furthermore, the minimum value of the energy release rate is obtained for the large thickness of the steel adherend.

## 5. Discussion

The parametric study, conducted using the simplified analytical Interface Fracture Mechanics (IFM) methodology, provided key insights into how geometric variables influence the crack driving force ($G_{int}$) and fracture mode mixity (ψ) at the zinc–adhesive interface in single-lap joints. This analysis allows for a deeper interpretation of the experimental results reported by Dybizbański et al. (2024) [26].

### 5.1. Model Simplifications and Limitations

The analytical methodology employed in this study was intentionally simplified to serve as a tool for rapid, qualitative assessment of parametric trends. It is crucial, however, to acknowledge the limitations stemming from these simplifications. The most significant approximation is the use of constant, general-purpose shape functions to calculate the stress intensity factors. In reality, these functions are complex and depend on the relative crack length (a/t_a) and the elastic mismatch between the materials, as described by the Dundurs parameters. The adopted constant values are classical solutions for edge cracks in homogeneous materials under specific conditions and do not capture the full complexity of the interfacial stress field [22]. This choice inherently limits the quantitative accuracy of the calculated energy release rate values. Therefore, the results of this model should be interpreted as indicative of qualitative trends rather than as precise numerical predictions. For enhanced quantitative accuracy, a finite element-based calibration of the shape functions for the specific joint geometry and material combination would be a necessary and valuable next step.

A second important simplification is the treatment of the thin (≈9 µm) zinc layer as a discrete, isotropic elastic material. Given that its thickness is orders of magnitude smaller than the adherend and adhesive dimensions, a more physically representative approach for a high-fidelity analysis would be to model it as a compliant interphase or using a gradient property model. Such advanced models, often employing cohesive zone elements, can better capture the specific compliance and potential failure mechanisms within the coating itself. The first-order approximation used in this study was chosen for its consistency with the overall simplified analytical framework, which assumes that failure occurs perfectly at the zinc–adhesive interface. Investigating more sophisticated interphase models remains a compelling and important avenue for future research.

### 5.2. Analysis of Experimental Fracture Toughness Data

The significant scatter observed in the experimental interfacial fracture toughness data, as plotted in Figure 3, is a key finding that warrants further discussion. This variability, while substantial, is not unexpected and can be attributed to a combination of factors related to the material’s microstructure, the adhesive bonding process, and the idealizations within the analytical model. It is important to note that the data points correspond exclusively to specimens that exhibited failure at the zinc–adhesive interface, as verified by post-failure visual inspection [26].

A primary source of this scatter is the complex, non-homogeneous nature of the hot-dip galvanized zinc layer itself. The coating is not a monolithic material but a multi-phase system comprising several brittle zinc–iron intermetallic phases (such as Γ and δ phases) beneath the outer pure zinc layer [35]. The fracture behavior and damage mechanisms within the zinc coating are highly sensitive to these microstructural features [36,37]. For instance, research has demonstrated a direct relationship between the fracture toughness of zinc coatings and their grain boundary distribution, with different boundary types exhibiting varied cracking resistance [38]. Consequently, the initiation of a crack at the zinc–adhesive interface is not governed by a single material property but is highly dependent on these local microstructural variations, which are a significant source of the scatter observed in the macroscopic joint strength.

Furthermore, such variability is a well-documented characteristic of adhesive joint testing in general. Extensive reviews of adhesion science have shown that high coefficients of variation, often between 20% and 50%, are common due to a multitude of factors, including microscopic voids, non-uniformities in the bondline thickness, and subtle variations in the curing process [39]. The apparent strength of a joint is also highly dependent on the bending stiffness of the adherends, which directly affects the peel stress distribution and can introduce further variability [40]. Finally, the simplifications inherent in the analytical model, particularly the use of constant shape functions as discussed in Section 5.1, contribute to the calculated scatter by not capturing the full complexity of the stress state at the crack tip for each specific specimen. Considering these factors, the plot should not be interpreted as a precise, single-valued failure locus but rather as an estimation of the range of interfacial fracture toughness for this specific material system under the given test conditions.

The relationship presented in Figure 3, with the addition of a trendline, allows for a more nuanced interpretation of these results. The underlying parabolic trend suggests a fundamental, mechanics-driven relationship between fracture toughness and mode mixity, which is consistent with IFM theory. This indicates that while local material inconsistencies and experimental variability dictate the precise failure load of any single specimen, the average behavior of the interface is governed by the phase angle, with a clear peak in toughness under a specific mixed-mode condition. Therefore, the plot should be interpreted as showing two coexisting phenomena: a deterministic, mechanics-based trend (the parabola) superimposed with stochastic, material- and process-induced variability (the scatter).

### 5.3. Effect of Adhesive Thickness on Fracture Mode

The parametric study indicates a clear trend where an increase in adhesive thickness shifts the fracture mode towards being more opening-dominated, as reflected by the rising phase angle ψ. While this result is derived from a simplified analytical model, the underlying physical mechanism is well-established in the literature on single-lap joints (SLJs). The eccentric load path inherent in the SLJ configuration induces joint rotation, which in turn generates significant peel stresses (the Mode I component) at the edges of the overlap—a phenomenon extensively analyzed since the work of Hart-Smith [41].

A thicker adhesive layer results in a more compliant bondline. This increased compliance allows for greater joint rotation under load, which amplifies the bending moment in the adherends and, consequently, the magnitude of the peel stresses at the bond ends [42]. This effect often leads to a reduction in the overall strength of the joint as the adhesive thickness increases [43]. This shift in the local stress state, with a higher contribution from Mode I, naturally favors a more opening-dominated fracture. Indeed, several studies have confirmed that as the bond thickness increases, the entire fracture mechanism within the adhesive joint can change completely [44]. Therefore, the qualitative trend captured by the presented model is a correct reflection of the fundamental mechanics of single-lap joints, corroborated by extensive experimental and numerical research.

### 5.4. Comparison of Parametric Trends with the Existing Literature

The parametric trends identified in this study, despite being derived from a simplified model, show strong agreement with the findings reported in more complex experimental and numerical investigations of CFRP-steel bonded joints. A significant body of research, including the work of Zeng et al. (2018) [24] and Teng et al. (2012) [22], has focused on debonding failures in large-scale CFRP-strengthened steel structures, establishing fracture mechanics as the essential framework for analyzing interfacial behavior. While these studies often involve more complex geometries, the fundamental mechanical principles governing the influence of joint parameters are consistent with the trends observed in the presented analysis.

Presented finding that increasing the overlap length reduces the energy release rate ($G_{int}$) for a constant load is consistent with the well-known principle that stress concentrations in single-lap joints are localized at the ends of the overlap. For longer overlaps, a central portion of the bondline remains practically unloaded, which effectively distributes the load and reduces the crack driving force at the joint’s edge [45]. This explains the observed plateau in $G_{int}$ for longer overlap lengths in the presented analysis and aligns with findings that joint strength does not increase proportionally with overlap length, especially for brittle adhesives.

Similarly, the model’s prediction that a thicker adhesive layer increases $G_{int}$ and shifts the mode mixity towards opening-dominated fracture (a higher phase angle ψ) is strongly supported by the literature. As detailed in Section 5.3, this phenomenon is attributed to increased joint rotation and amplified peel stresses, a trend widely reported in studies on metal–composite joints [41,42]. The consistency of presented results with the broader literature findings confirms that the simplified analytical model, while not quantitatively precise, successfully captures the correct qualitative relationships between key geometric parameters and the governing fracture mechanics parameters. This validates its utility as a tool for preliminary research and design exploration and for interpreting experimentally observed failure trends.

Furthermore, the parametric trends predicted by the model provide a direct, mechanical explanation for the key experimental observations reported in. The experimental study found that joint strength decreased with increasing adhesive thickness and generally increased with longer overlaps. The model aligns perfectly with these findings on a qualitative basis: a thicker adhesive increases the crack driving force ($G_{int}$) for a given load (Figure 5), which means the critical fracture toughness is reached at a lower external load, thus reducing strength. Conversely, a longer overlap decreases $G_{int}$ (Figure 4), requiring a higher applied force to initiate failure. This strong consistency confirms the model’s utility in capturing the fundamental mechanisms governing the joint’s behavior and enhances the interpretation of the experimental results.

## 6. Conclusions

This paper presented a parametric analysis of interfacial cracking in steel–CFRP single-lap joints using a simplified IFM methodology, contextualized with experimental data. The analysis leads to the following key conclusions:

- The experimental data, when analyzed with a trendline, suggests a non-linear, parabolic relationship between the interfacial fracture toughness ($G_{int}$) and the phase angle (ψ). The interface exhibits a peak fracture toughness at a phase angle of approximately ψ≈11.5°, indicating that the joint’s resistance to cracking is highest under a specific mixed-mode condition. The significant scatter around this trend highlights the influence of local microstructural variations and process-induced variability.
- The parametric model demonstrates that for a constant load, increasing the overlap length reduces the crack driving force ($G_{int}$), while increasing the adhesive thickness raises it. These trends provide a direct mechanical explanation for experimental observations where longer overlaps increased joint strength and thicker adhesives decreased it, thus validating the model’s qualitative predictive capabilities.
- A key finding, strongly supported by established joint mechanics, is that a thicker adhesive layer shifts the fracture mode from shear-dominated to opening-dominated (a higher phase angle ψ). This is attributed to the increased joint rotation that amplifies peel stresses at the bond ends.
- The study confirmed the model’s sensitivity to the assumed initial crack length, with $G_{int}$ being directly proportional to it, which is characteristic of LEFM-based analyses. Furthermore, increasing the thickness of the steel adherend was found to consistently reduce the crack driving force across all analyzed geometric parameters.
- In summary, the simplified analytical model, despite its acknowledged limitations—primarily the use of constant, general-purpose shape functions—effectively captures the qualitative influence of key geometric parameters on the joint’s fracture behavior. It provides a sound mechanical basis for interpreting experimentally observed failure trends and serves as a useful, resource-efficient tool for preliminary research and design explorations.

## Data Availability

The original contributions presented in this study are included in the article. Further inquiries can be directed at the corresponding author.
